# Interfacial and confined molecular-assembly of poly(3-hexylthiophene) and its application in organic electronic devices

**DOI:** 10.1080/14686996.2022.2125826

**Published:** 2022-09-27

**Authors:** Junhao Liang, Xing Ouyang, Yan Cao

**Affiliations:** a Advanced Institute for Soft Matter Science and Technology (AISMST), School of Emergent Soft Matter, South China University of Technology, Guangzhou, China; b College of Materials Science and Engineering, Shenzhen University, Shenzhen, Guangdong, China; c Guangdong Provincial Key Laboratory of Functional and Intelligent Hybrid Materials and Devices, South China University of Technology, Guangdong, China

**Keywords:** P3HT, interfacial assembly, confinement, nanomaterials, organic electronic device

## Abstract

Poly(3-hexylthiophene) (P3HT) is a typical conducting polymer widely used in organic thin-film transistors, polymer solar cells, etc., due to good processability and remarkable charging carrier and hole mobility. It is known that the ordered structure assembled by π-conjugated P3HT chains could promote the performance of electronic devices. Interfacial and confined molecular-assembly is one effective way to generate an ordered structure by tuning surface geometry and substrate interaction. Great efforts have been made to investigate the molecular chain assembly of P3HT on a curved surface that is confined to different geometry. In this report, we review the recent advances of the interfacial chain assembly of P3HT in a flat or curved confined space and its application to organic electronic devices. In principle, this interfacial assembly of P3HT at a nanoscale could improve the electronic properties, such as the current transport, power conversion efficiency, etc. Therefore, this review on interfacial and confined assembly of P3HT could give general implications for designing high-performance organic electronic devices.

## GRAPHICAL ABSTRACT

**Figure:**
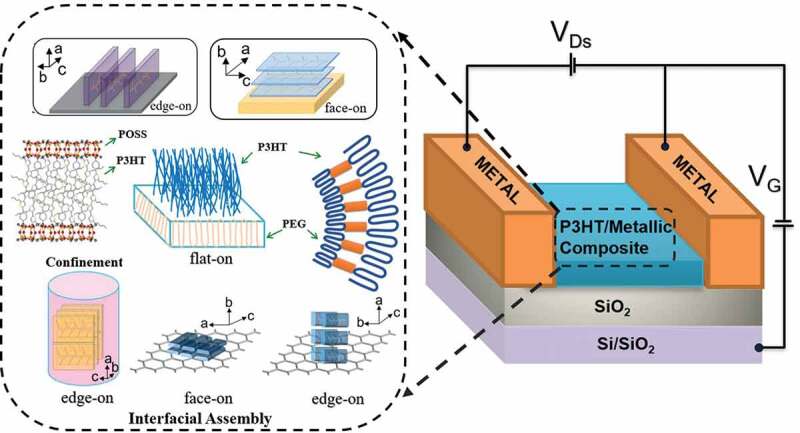

## 1. Introduction

Chain orientation and packing of π-conjugated polymers greatly affect the properties of functional electronic devices. Poly(3-hexylthiophene) (P3HT) is a common conducting polymer and has been widely used in function electronic devices [1–12]. The main backbone of P3HT is composed of thiophene rings and the side group is short alkyl chains. Two main forms exist in thiophene polymers (form I, *a* = 1.6 nm, *b* = 0.77 nm, *c* = 0.76 nm, *α* =*β* = 90°, *γ* = 87°; form II, *a* = 1.076 nm, *b* = 0.777 nm, *c* = 0.944 nm, *α* = *γ* = 90°, *β* = 64.66°) [13,14].

Confinement is one of the efficient methods to manipulate the chain-assembly at a molecular level. Both the geometry of confined space and interfacial interaction plays an important role in assembling the ordered structure of functional molecular chains [15–30]. A lot of research have reported novel nanostructures like nanotwin or coherent crystal branching and so on could be produced under cylindrical confinement. Meanwhile, the π-π stacking orientation of a conducting polymer is dependent on the interaction between the conductive polymer and the substrate. Furthermore, P3HT could be fabricated into several kinds of nanostructures, such as nanotubes, nanowires, thin films and 2D-sandwich nanocrystals [31–49]. It has been found that in the ordered and well-aligned chain-assembling structure of P3HT nano-devices, the hole and the charge carrier mobility transport is more efficient [50–60].

In this report, we review the recent advances in interfacial and confined chain-assembly of P3HT nanostructure in different types of substrate and its application in functional and organic electronic devices, such as organic field effect transistors (OFET), organic photovoltaic (OPV), organic light-emitting diodes (OLED) and etc [57,61–80]. The effect of temperature, confined spatial geometry, substrate surface, and interface on the functional P3HT nanomaterial has been described below:

## 2. π-π stacking and chain orientations of P3HT on the substrate

**Figure 1.**
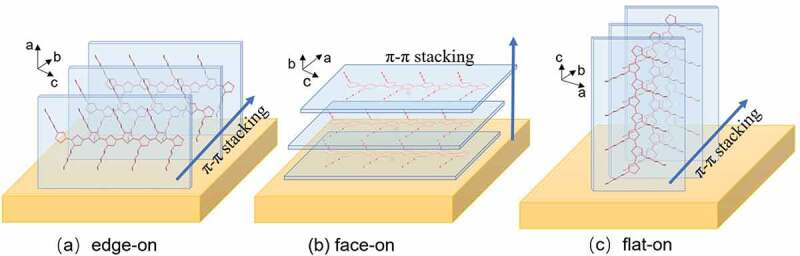
Schematicsof (a) *edge*-on, (b) *face*-on, (c) *flat*-on π-π stacking and chain orientation of P3HT films on the substrate.

According to Agbolaghi et al., there are three main π–π stacking orientations of the lamellar in P3HTgenerated on the substrate as shown in Figure 1: the edge-on orientation (the thiophene-backbone plane is vertical to the substrate), the face-on orientation (the thiophene-backbone plane is parallel to the substrate), the flat-on orientation (the thiophene-backbone plane, as well as the molecular chain orientation, are vertical to the substrate) [81–83].

## 3. Interfacial assembly of P3HT and its application in functional electronic devices

### 3.1. The rubbing effect on the lamellar orientation of P3HT in thin film

**Figure 2.**
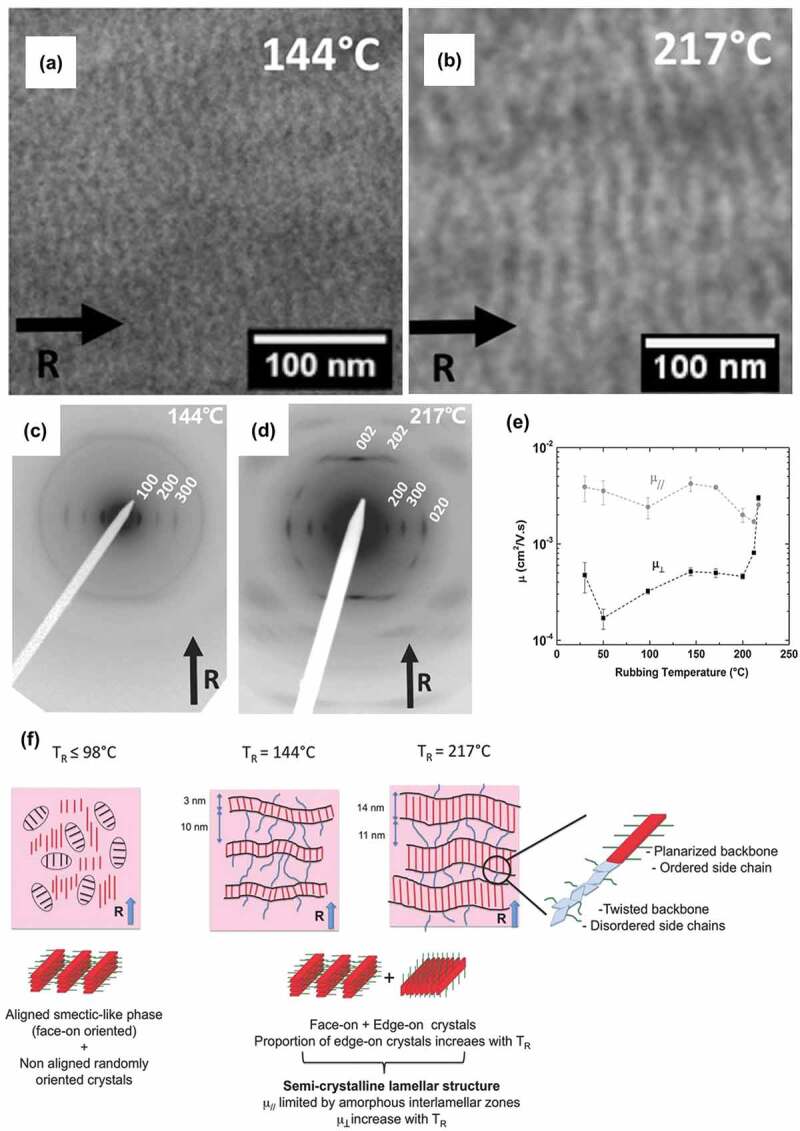
TEM bright-field images of thediv div morphologies in oriented P3HT films prepared by mechanically rubbing at (a)144 ℃ and (b) 217 ℃, respectively.div div ED patterns of P3HT films when rubbed at (c) 144 ℃, (d) 217 ℃; (e) the curves of hole mobility in bottom-gate/bottom-contact OFETs made by rubbing P3HT films. μ// and μ⊥ represents the hole mobility is measured by parallel or perpendicular to the rubbing direction; (f) Schematic illustration of the lamellar orientation’s changes with respect to the temperature. Reprinted from Ref. [82] with permission. Copyright 2016.

Hamidi-Sakr et al. investigated the crystal orientation of regioregular P3HT thin films prepared by mechanical rubbing at a temperature in the range 144–217℃ [82]. Figures 2(a,b) display the alternated black and white line morphology of thin films of P3HT prepared by mechanically rubbing at 144℃ and 217℃, respectively, indicating that periodic lamellar morphology is formed and becomes more regular at high temperatures. The lamellar period, *l*, turns to be bigger when increasing the rubbing temperature. *L* improved from 13 to 26 nm when the temperature changes from 144℃ to 217℃. The selected-area electron diffraction patterns of transmission electron microscopy (TEM) of P3HT thin film crystals prepared at 144℃ and 217℃ are displayed in Figure 2(c,d), respectively. According to the literature report, the (020) reflection at the equator stands for the edge-on lamellae orientation, while the equatorial reflection of (*h*00) represents the face-on orientation. The schematic illustration of the crystal orientation change in rubbed P3HT film with respect to the temperature is shown in Figure 2(f). Below 98℃, the films exhibited a highly strained smectic-like phase with the thiophene backbones extending along the rubbing direction, implying the face-on orientation has been formed. But when the rubbing temperature was increased from 140 to 217℃, both the h00 reflections and the 020 reflection are located at the equator. Therefore, both face-on and edge-on orientations coexist in the 144–217℃.

Kajiya and Saitow et al. have investigated the effect of rubbing on the photoconductivity of three P3AT-based compounds: P3DDT, P3HT and P3BT with different alkyl side chain length which are –C_12_H_25_, –C_6_H_13_ and –C_4_H_9_ respectively. It is obvious that the film rubbing has significantly improved the out-of-plane mobility of the three P3AT-based samples that were identified by polarized electronic absorption spectroscopy and 2D grazing-incidence X-ray diffraction [84,85]. On the other hand, when the P3HT was applied in organic solar cell, they found that the out of plane mobility was 8 orders enhanced by rubbing in low regularity P3HT samples. This is because the more amount of face-on orientation has been formed during the rubbing process and thus more π−π stacking orientation of P3HT inclined to be the out-of-plane direction instead of in-plane direction.

In summary, the lamellar stacking orientation of P3HT can be manipulated by mechanically rubbing the films at different temperatures. Furthermore, besides the temperature impact of the packing structure, other factors such as molecular weight, the solvent used for the film preparation and etc. also affect the lamellar orientation [78,83,86–91].

### 3.2. Interfacial interaction and confinement effect on the molecular assembly on the (reduced graphene oxide) RGO surface

Agbolaghi et al. investigated that the thiophene rings of P3HT interact with the bared surface of RGO, causing a conventional face-on orientation. In this case, the molecular chains of P3HT are parallel to the RGO surface. For comparison, the functionalized-RGO could interact with the side chains of P3HT and therefore generate edge-on orientations. Meanwhile, the researchers also noticed that with increasing the crystallization time, the fibrils of P3HT tend to be parallel to the bared-RGO surface. And under these circumstances, the chain orientations are perpendicular to the RGO surface normal [92].

**Figure 3.**
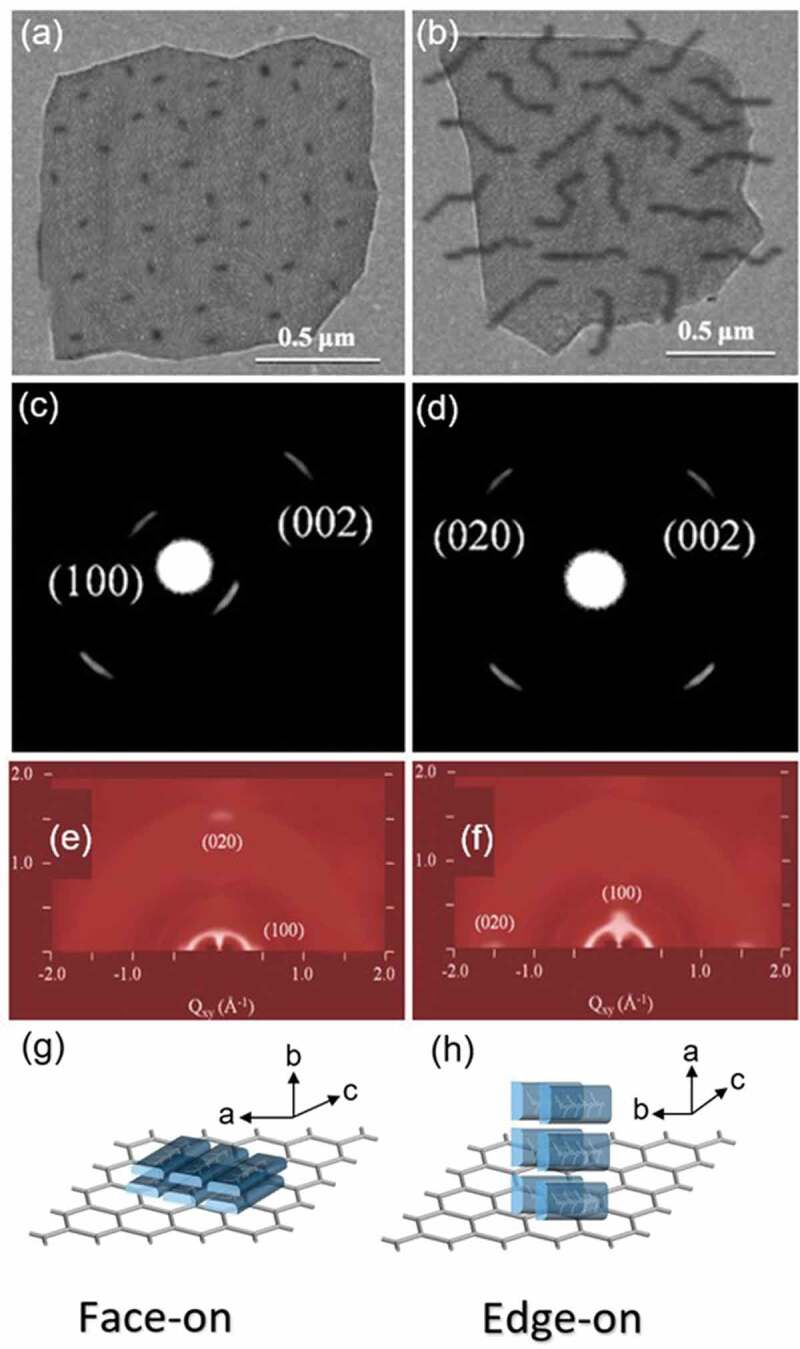
TEM BF images for P3HT crystals grown on the graphene in toluene about (a) 6 *h*, (b) 18 *h*; (c) selected-area electron diffraction pattern of the P3HT crystals (face-on) grown on the graphene about 6 *h*; and (d) selected-area electron diffraction pattern of the P3HT crystals (edge-on) grown on the graphene about18 *h*; GIWAXDpatterns of P3HT on grown intoluene about (e) 6 *h*, (f) 18 *h*; schematic illustration of P3HT crystals with (g) face-on and (h) edge-on orientation on the graphene substrate. Reprinted from Ref. [92] with permission. Copyright 2018.

Figure 3(a,b) describe the TEM bright-field (BF) image of P3HT crystals grown on the RGO surface for 6 h and 18 h, respectively. Figure 3(c,d) display the [010] and [100] zone electron diffraction patterns of P3HT crystals that are grown on the RGO surface, respectively. Figure 3(e,f) show the grazing-incidence wide-angle X-ray diffraction (GIWAXD) patterns of P3HT crystals isothermally crystallized on the RGO surface for 6 hrs and 18 hrs, respectively. Both electron diffraction (ED) patterns and GIWAXD patterns show that *b*-axis ((010) direction) in the face-on assemblies is oriented normal to the RGO surface when the P3HT is crystallized on the RGO surface in a shorter time (6 hours). On the other hand, *a*-axis ((100) direction) in the edge-on assemblies is perpendicular to the RGO surface when the P3HT is crystallized on the RGO surface for a longer time (18 hours). The reason for the change of lamellar orientation in P3HT with respect to the crystallization time can be described as follows: At the initial stage of crystallization of P3HT on the RGO surface, the interactions between the thiophene-rings and phenyl-rings of RGO favor both the plane of thiophene-ring and molecular chains inP3HT are parallel to the RGO surface. Therefore, the π-π stacking orientations are directed along the surface normal and face-on orientation is generated. However, for the later stage (longer time) in crystallization, the 1D confinement imposed by the thin film’s thickness on the RGO surface prevails, and the fast growth direction (the *b*-axis) of P3HT fibrils is aligned perpendicular to the surface normal and thus induces an edge-on orientation. In this case, the plane of the thiophene-ring is perpendicular to the RGO surface.

Another important issue is that the interfaction interaction between the conjugated polymers and substrate could affect the chain conformations and its packing structure in P3HT aggregation. It is known that for conjugated polymers, H- and J-aggregates are composed of the extended chain and folded chain, respectively. And the microstructure of the lamellar aggregates could affect the hole mobility and thus the device performance [93,94]. Normally, the aggregate of conjugated polymer is a mixture of H- and J-aggregate. Recently, an ultrapure 99.8% H-aggregate polymer can be obtained by casting a droplet of P3HT solution on a low-wettability substrate at room temperature. The hole mobility of nearly pure H-aggregate is a 6-fold increase compared to a common P3HT film with a mixture of H- and J-aggregate.

In addition, when using the solution crystallization process to prepare P3HT films, the solvent selection of the solution is an important factor affecting the film structure. Recently, many studies have reported the effect of solvent on the molecular orientation of conjugated thin films [95,96]. It has been found that the molecular chain orientation of P3HT in perovskite films for solar cells can be manipulated by using organic solvents with specific volatile properties.

### 3.3. P3HT chains/nanosheets self-assembling at the air/liquid interface

**Figure 4.**
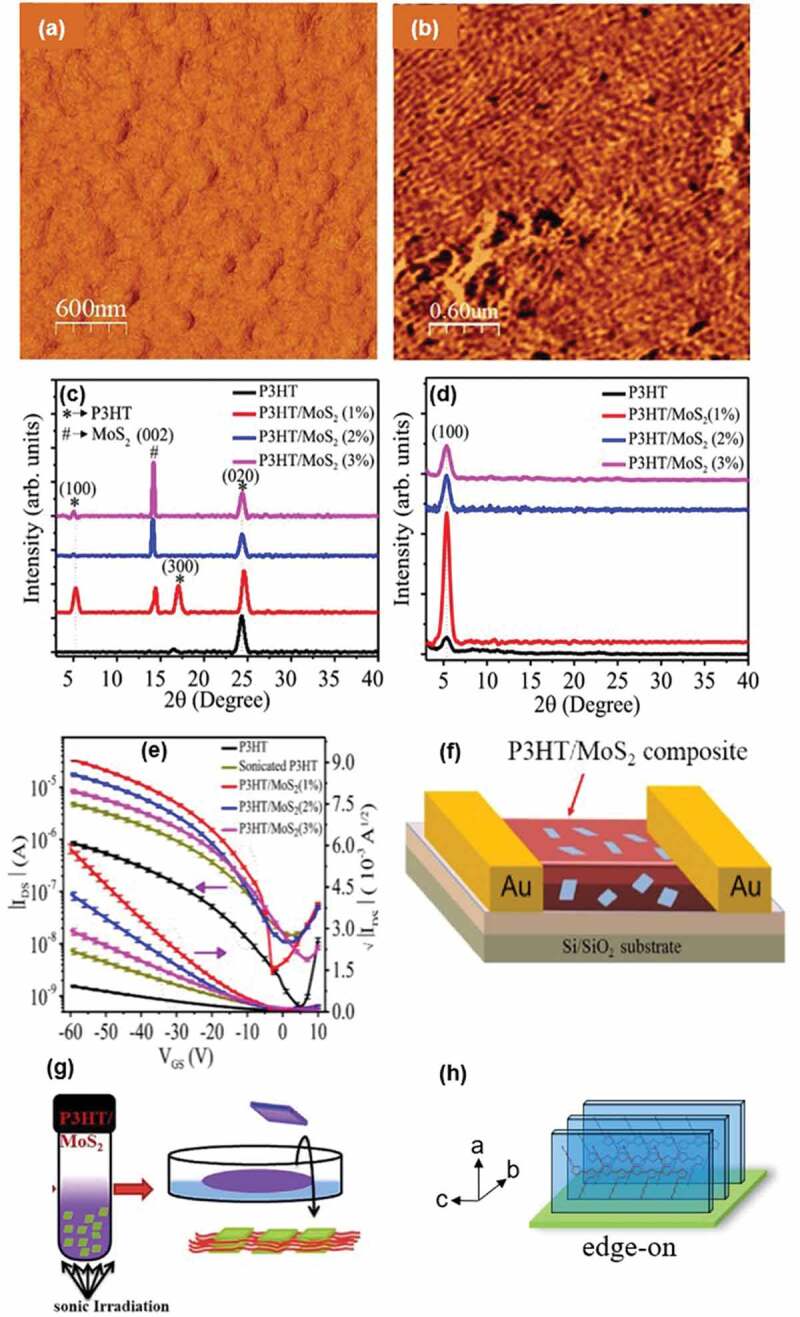
AFM phase image of (a) pristine P3HT and (b) P3HT/MoS_2_ (1%);(c) powder XRD and(d) GIWAXD pattern of pure P3HT and P3HT/MoS_2_ with variousratios (1%, 2% and 3%); (e) transfer characteristics of pristine P3HT, sonicated P3HT and P3HT/MoS_2_ (1%, 2% and 3%) OFETs with standard deviations; (f) schematic illustration of OFET devices made by P3HT/nanosheets; (g) fabrication process of P3HT/MoS_2_ nanocomposite under ultrasonic treatment; (h) edge-on orientation of dP3HT/MoS_2_ nanocomposite. Reprinted from Ref. [97] with permission. Copyright 2020.

To achieve highly efficient charge transport in organic electronic devices, the long-range ordering structure of π-conjungated polymer crystals is desirable (Figure 4). *Chaudhary et al*. prepared a uniform thin film of P3HT/MoS_2_ via surface-tension gradient and compressive force. The thin film is formed by casting the solution containing the ultrasonicated P3HT/MoS_2_ nanocomposite onto the liquid surface of a glycerol and ethylene glycol mixture shown in Figure 4(g) [97]. The phase morphology of pristine P3HT (Figure 4(a)) and P3HT/MoS_2_ (1%) nanocomposite (Figure 4(b)) has been studied by atomic force microscopy (AFM). It is obvious that compared to the phase image of pristine P3HT, long fibril crystals are formed and aligned almost in one direction as shown in the phase images of P3HT/MoS_2_ (1%) nanocomposite and MoS_2_ nanosheets are also included in the thin film. The well-aligned and ordered structure of P3HT fibril crystals and MoS_2_ nanosheets are generated by the surface tension gradient between the polymer solution and liquid substrate (Figures 4(a,b)).

The ordered structure of P3HT/MoS_2_ (1%) nanocomposite has been examined through GIWAXD and X-ray powder diffraction experiments (Figures 4(c,d)). The equatorial (020) reflection represents the edge-on orientation of P3HT crystals formed on the substrate in P3HT/MoS_2_ (Figure 4(h)). As for the charge transport properties in the P3HT/nanocomposites, the typical drain current/back-gate voltage (*I*_DS_-*V*_DS_) curves indicate it has a *p*-type charge transport (Figure 4(e)). The drain current of P3HT/MoS_2_ is larger than that of pristine P3HT. The P3HT/MoS_2_ (1%) possessed the maximum degree of electronic mobility because the edge-on orientation brings in the lowest exciton bandwidth and longest conjugation length compared to other samples. In addition, when increasing the amount of MoS_2_, the electron mobility decreases. Because a large number of nanosheets prohibits the growth of P3HT fibril crystals and thus, the length of fibril crystals is reduced [98,99].

Furthermore, according to the literature, the hole mobility and density of Si-nanocrystal/P3HT hybrid film have been greatly increased about 50- and 12-fold of the pristine P3HT, respectively, due to higher crystallinity of hybrid P3HT film induced by homogeneous distribution Si-nanocrystals. The hole moves to be parallel to the out-of-plane direction of Si-nanocrystal/P3HT film or film surface normal. Meanwhile, the inorganic Si-nanocrystals can improve film stability [100].

The Langmuir-Blodgett (LB) method is another important way to fabricate the highly ordered film of conjugated polymers at liquid/air interface. And the stable and well-defined LB film on conjugate polymer can be transferred onto the hydrophobic substrate and thus can be applied for constructing organic devices, such as FET and etc [101–103]. For the uniform LB film of regioregular P3HT, in most cases, the backbone of P3HT is parallel to the substrate surface and the preferred molecular orientation is in an edge-on orientation. The high-filed effect mobility (2.2 × 10^−4^ and 4.4 × 10^−4^ cm^2^ V^−1^ s^−1^) for mixed regioregular P3HT were obtained and used as the semiconducting film in the OFET.

### 3.4. 1D confined chain-assembly of P3HT in the 2D polyhedral oligomeric silsesquioxanes (POSS)-layered nanocrystals

**Figure 5.**
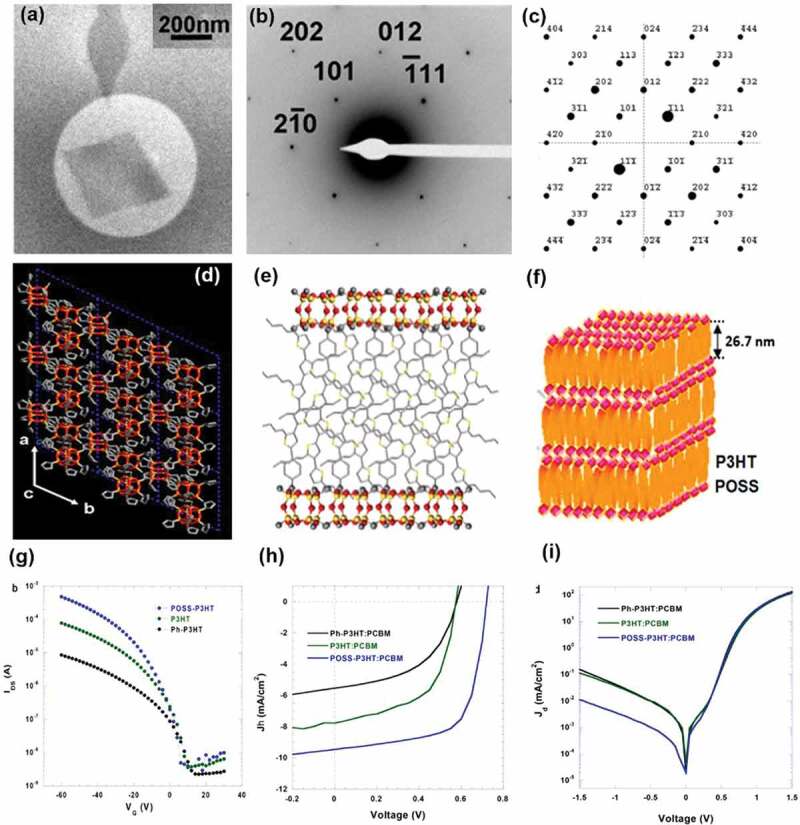
(a) TEM BF morphology of the 2D crystals (POSS-P3HT); (b) selected-area electron diffraction pattern of POSS-P3HT single crystal; (c) calculated diffraction pattern with the [12$\overset{ˉ}{1}$] zone; (d) *ab*-plane projection of 2D crystals of POSS-P3HT; (e) schematic illustration of the molecular packing of the 2D crystals (POSS-P3HT); (f) hierarchical structure of the 2D crystals (POSS-P3HT); (g)*I*_*DS*_ versus *V*_*G*_curves of thin flim transisitors prepared by 2D nanocrystals (POSS-P3HT), pristine P3HT and Phenyl-P3HT J – V characteristics of thin film transistors preparedfrom 2DPOSS-P3HT nanocrystals, pristine P3HT and Phenyl-P3HT thin films (h) with and (i) without white illumination. Reprinted from Ref. [106] with permission. Copyright 2019.

Two-dimensional materials can be designed to improve the charge-carriers mobilities, mechanical, electronic, and optical properties. *Taoet al*. found a layered 2D nanocrystals of POSS-P3HT can be applied to the organic thin film transistor. The POSS-P3HT synthesized with click chemistry contains one P3HT molecular chain covalently linked with one POSS molecule [104,105]. The TEM bright-field morphology is displayed in Figure 5(a) and the selected-area electron diffraction experiments corresponding to the crystal in Figure 5(a) are shown in Figure 5(b). The simulated pattern with the [12$\overset{ˉ}{1}$] zone of the 2D crystal on POSS-P3HT is displayed in Figure 5(c). The 2D POSS-P3HT crystals own a hexagonal unit cell with *a* = *b* = 1.606 nm, and *c* = 1.714 nm with a symmetry group of P6. Based on the electron diffraction and wide-angle X-ray diffraction results of 2D crystals, we established the molecular packing model of 2D crystals(Figures 5(d–f)). The P3HT chains interdigitated between two crystalline layers of POSS [106]. The sandwich 2D crystal structures could be also confirmed by the one layer’s thickness (26.7 nm) measured by atomic force microscopy. It is worth mentioning that the power conversion efficiency of the POSS-P3HT 2D crystal is 40% higher than that of the pristine P3HT mixed with [6,6]-phenyl-C_61_-butyric acid methyl ester (PCBM) (Figures 5(g–i)).

### 3.5. Solution-Floating interfacial-assembly of P3HT nanowires

It is known that the evenness and morphology of P3HT thin films play a key role in the properties of functional electronic devices. *Kim et al*., invented a solution-floating self-assembly method to prepare the thin film of P3HT. P3HT samples are first dissolved into a toluene solution [107]. A droplet of the solution was subsequently cast onto the water surface and a thin film of P3HT could be generated once the toluene was completely evaporated. The morphology of P3HT nanostructures can be tuned by changing the different types of solvents. Compared to the spin-coating methods for thin film preparation, the solution-floating assembling methods have more advantages and could be used to form a more ordered structure with high crystallinity.

**Figure 6.**
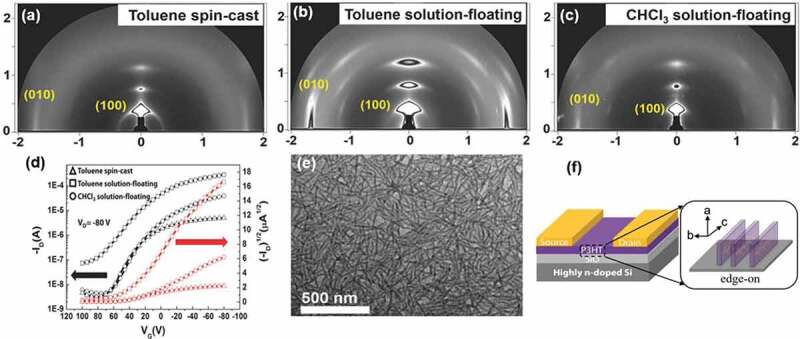
(a) TEM BF image and (b) ED pattern of scrolled half-rings crystals of P3HT_7000_-b-PEG_5000_ block copolymers isothermally crystallized at 30°C; (c) TEM BF image and (d) ED pattern of P3HT_7000_-b-PEG_5000_ square single crystals seedily grown at *T*_*c*_ = 30°C for 24 *h* in amyl acetate.(e) TEM BF image and (f) ED pattern of the fibrillar single crystals of pure P3HT; (g) 2D GIWAXD pattern of cubic P3HT-*b* PEG crystals isothermally crystallized at 30°C; (h) Schematic illustration of homopolymer-P3HT fibrillar crystal,p3ht-b-PEG scrolled half-rings crystals and flat-on crystals. Reprinted from Ref. [107] with permission. Copyright 2016.

The nanostructure and morphology generated in the thin film deeply rely on the solvent type for polymer and must be water-immiscible. Using the solution-floating methods, P3HT can self-assemble into a highly uniform thin film made up of nanowires. The GIWAXD results of the P3HT film prepared by toluene spin-cast, toluene solution-floating, and CHCl_3_ solution-floating are shown in Figure 6(a–c)), respectively. The equatorial *010* and meridian *h00* reflections manifest an edge-on orientation in the thin films of P3HT nanowires. Based on the transfer curves (*I*_*D*_–*V*_*G*_) of the OFET device, more ordered nanostructure and morphology could enhance the electronic properties of the organic electronic device. And the OFET device prepared by self-assembling P3HT via solution floating method has at least 3.4 times mobility value (0.086 cm^2^ V^−1^s^−1^) than that of assembling molecular chains of P3HT with spin-coating method (0.025 cm^2^ V^−1^s^−1^).

A well orientated P3HT film can also be prepared by soft friction transfer methods, which is an easy approach to fabricate an uniaxially oriented P3HT film at room temperature and ambient atmosphere [108]. And the film can be transferred from the glass substrate by using cellulose solution. This novel soft friction transfer method is a promising way to be used in next-generation transistors, sensors and other soft electronic devices.

### 3.6. Interfacial assembly of molecular chains in scrolled/flat crystals of P3HT

The crystal geometry of P3HT can be tuned by interfacial assembly of molecular chains in P3HT at a curved surface that are covalently linked with poly(ethylene glycol) (PEG) as block copolymer with different crystallization conditions [109].

**Figure 7.**
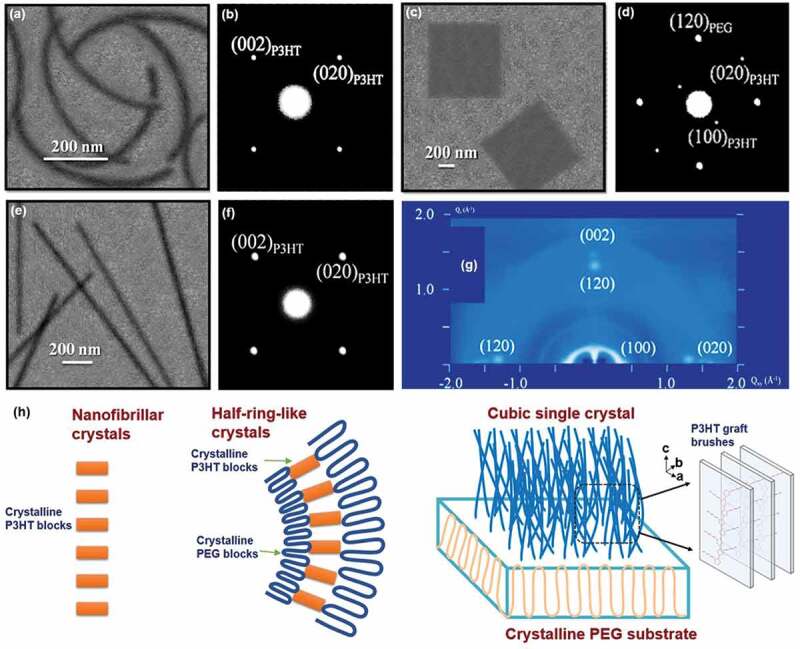
(a) TEM BF image and (b) ED pattern of scrolled half-rings crystals of P3HT_7000_-b-PEG_5000_ block copolymers isothermally crystallized at 30 °C; (c) TEM BF image and (d) ED pattern of P3HT_7000_-b-PEG_5000_ square single crystals seedily grown at T_c_ = 30 °C for 24 *h* in amyl acetate.(e) TEM BF image and (f) ED pattern of the fibrillar single crystals of pure P3HT; (g) 2D GIWAXD pattern of cubic P3HT-*b* PEG crystals isothermally crystallized at 30 °C; (h) Schematic illustration of homopolymer-P3HT fibrillar crystal, P3HT-b-PEG scrolled half-rings crystals and flat-on crystals. Reprinted from Ref. [109] with permission. Copyright 2016.

The half-ring crystals of P3HT shown in Figure 7(a) were observed in a solution crystallization of the pure P3HT and the P3HT-*b*-PEG at 30 ℃ without self-seeding. In this case, the sandwiched and curved crystal of P3HT can be formed in the middle. The inner and outerside of scrolled crystals of P3HT-*b*-PEG are PEG crystals and the packing density of PEG chains in both sides of middle P3HT crystals is not equal, and the unbalanced surface stress between inner and outside crystals results the curved crystal. The thickness of PEG lamellar on both sides was 17.4 (outer) and 15.0 (inner) nm. The scrolled crystals of P3HT are an edge-on orientation, identified by the 002 and 020 electron diffraction spots via TEM selected-area electron diffraction technique (Figure 7(b)) and an 020 equatorial orientation was displayed in the 2D GIWAXD experiments.

On the other hand, the square crystals of P3HT shown in Figure 7(c) can be obtained by a two-step crystallization process by adding the pure PEG seeds formed at 40 ℃ to crystallize P3HT_7000_-b-PEG_5000_ solution at 30 ℃ via self-seeding technique. Four strong 120_PEG_ diffraction spots confirmed the single crystals of PEG are generated with the [001] zone (Figure 7(d)). 020_P3HT_ and 100_P3HT_ diffraction spots in the ED pattern (Figure 7(d)) reflects that P3HT crystals are epitaxially grown at the lamellar surface of PEO crystals. And the *c*-axis of the P3HT crystals is parallel to the PEG-lamellar surface normal. Therefore, under these circumstances, the P3HT crystals have an edge-on orientation. Generally speaking, the fibrillar crystals of P3HT (Figure 7(a)) can be obtained when crystallizing the pristine P3HT samples. The long-axis of fibrillar crystals is along the *b*-axis (Figure 7(b)). Overall, the schematic illustration of P3HT crystals with crystal geometry (fibrillar, scrolled and square) via interfacial assembly are shown in Figure 7(h).

### 3.7. Assembling the molecular chain of P3HT under geometrical (cylindrical) confinement and its application in organic solar cell device

**Figure 8.**
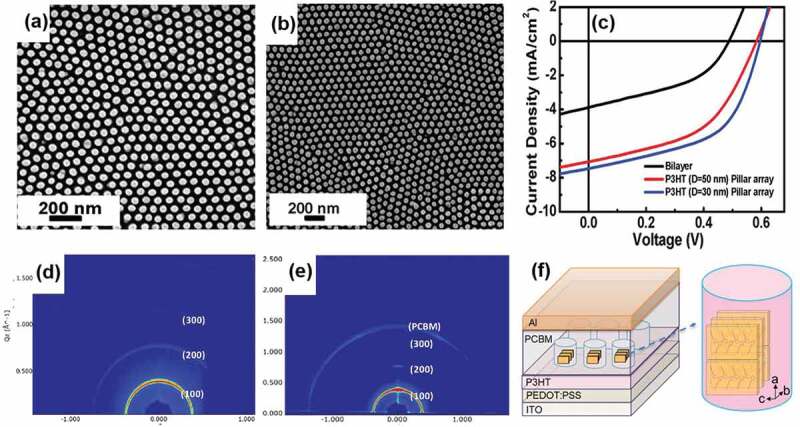
Scanning electron micrograph of (a) pure P3HT nanopillars; (b) P3HT/PCBM nanopillars; (c) *J-V* curves of the organic photovoltaic devices made by P3HT nanopillar; GIWAXD patterns of (d) pure P3HT nanopillars and (e) P3HT/PCBM nanopillars; (f) schematic illustration of organic solar cell devices with P3HT nanopillars; (g) the edge-on orientation of P3HT thin films. Reprinted with permission from Ref. [110]. Copyright 2012.

A mold-releasing agent (low molecular weight (MW) polydimethylsiloxane) was used to modify the template surface to release the nanorods easily from the template. Therefore, perfect free-standing nanorods of P3HT without collapsing as shown in Figure 8(a,b) can be fabricated by reducing the interaction between P3HT and nanoporous alumina [110]. Compared to the normal 2D confinement without modifying the template surface, the low MW polydimethylsiloxane was used to lower the surface energy of the template, and thus the rods are easily obtained after the template was etched by 5% KOH [111]. Figure 8(c) displays the *J-V* curves of the OPV devices fabricated by P3HT nanopillar (50 and 30 nm). Compared to the common thin film device of P3HT, the power conversion efficiency of the P3HT nanorod device can be improved by about 2%. The rod diameter was dependent on the pore diameter of the AAO templates. The P3HT nanorods with the aspect ratio and spacing of 30 nm rod are about 6.6 and 60 nm, respectively. The edge-on orientation of P3HT has been identified in both pure P3HT and P3HT/PCBM nanopillars for 50 nm (Figure 8(d,e). The molecular chains of P3HT are perpendicular to the rod long-axis. The ordered P3HT nanostructure induced by 2D confinement provides a new route to design the high-performance organic electronic device.

It is worth mentioning that the periodic P3HT surface nanostructure can be fabricated by laser radiation with a fluence of about 22～30 mJ/cm^2^. When this periodic surface structure of P3HT induced by laser radiation is applied in an organic solar cell acting as a bilayer covered by C_71_-butyric acid methyl ester, an enhancement of about 10% is found in the open-circuit voltages for the solar cell. And the interesting heterogeneous electrical conductivity of the laser-induced nanostructured solar cell does not affect the production of photocurrent [112].

## 4. Summary and perspective

The interfacial assembly of P3HT and its application in organic electronic devices has been summarized in this review. Four main factors greatly affect the molecular packing of P3HT at the interface: *a*. the interaction between P3HT and the substrate; *b*. the geometrical confinement; *c*. covalently linked with the nanoatoms or other polymer; *d*. thin film preparation methods. An edge-on orientation of P3HT can be obtained by self-assembling P3HT-based block copolymers, in which P3HT is chemically linked with a nanoatom such as POSS or the other polymer like PEG etc. In the 2D crystals of P3HT-based block copolymers, the P3HT crystals have a flat-on orientation on the substrate of 2D crystals. The geometrical (cylindrical) constraints on the crystallization of P3HT nanorods favor an edge-on orientation. Specific thin film preparation methods like using mechanical force or solution-floating self-assembling nanowire or spinning-coating thin film methods usually generate an edge-on orientation, which could be widely applied in organic field-effect transistors. For comparison, the interaction between thiophene rings and graphene promotes the face-on orientation, which can be used to improve the device efficiency of the organic solar cell.

Overall, understanding the formation mechanism of interfacial molecular self-assembly of P3HT and their relationship associated with the edge-on and face-on orientation is a key issue to manipulate the electron and hole to undergo orderly transporting, which has a deep impact on improving the performance of the organic electronic device.
