# Correction

**DOI:** 10.1080/14686996.2022.2139100

**Published:** 2022-11-01

**Article title**: Interfacial and confined molecular-assembly of poly(3-hexylthiophene) and its application in organic electronic devices

**Authors**: Junhao Liang, Xing Ouyang & Yan Cao

**Journal**: *Science and Technology of Advanced Materials*

**Bibliometrics**: Volume 23, Number 1, pages 619–632

**DOI**: https://doi.org/10.1080/14686996.2022.2125826

When the above article was first published online, the captions to Figures 2, 4 and 6 contained errors. The online version has now been corrected, and the correct figure captions can be found below:

**Figure 2**. TEM bright-field images of the oriented P3HT films prepared by mechanically rubbing at (a) 144 °C and (b) 217 °C, respectively. ED patterns of P3HT films when rubbed at (c) 144 °C, (d) 217 °C; (e) the curves of hole mobility in bottom-gate/bottom-contact OFETs made by rubbing P3HT films. μ// and μ⊥ represents the hole mobility is measured by parallel or perpendicular to the rubbing direction; (f) schematic illustration of the lamellar orientation’s changes with respect to the temperature. Reprinted from Ref. [82] with permission. Copyright 2016.**Figure 4**. AFM phase image of (a) pristine P3HT and (b) P3HT/MoS_2_ (1%); (c) powder XRD and (d) GIWAXD pattern of pure P3HT and P3HT/MoS_2_ with various ratios (1%, 2% and 3%); (e) transfer characteristics of pristine P3HT, sonicated P3HT and P3HT/MoS_2_ (1%, 2% and 3%) OFETs with standard deviations; (f) schematic illustration of OFET devices made by P3HT/nanosheets; (g) fabrication process of P3HT/MoS_2_ nanocomposite under ultrasonic treatment; (h) edge-on orientation of P3HT/MoS_2_ nanocomposite. Reprinted from Ref. [97] with permission. Copyright 2020.**Figure 6**. GIWAXD pattern of P3HT thin film prepared by (a) toluene spin-cast; (b) toluene solution-floating; (c) CHCl_3_ solution-floating; (d) *J*–*V* curves of P3HT thin films prepared by the methods as described in (a), (b) and (c); (e) TEM BF images of P3HT thin film made by solvent evaporation; (f) schematic illustration of OFET device fabricated by P3HT thin film with edge-on orientation. Reprinted from Ref. [107] with permission. Copyright 2017.

